# Risk of obstructive sleep apnea among health workers: results of a screening in a large Italian University Hospital

**DOI:** 10.1007/s00420-023-02029-9

**Published:** 2023-12-12

**Authors:** Gianluca Spiteri, Maria Grazia Lourdes Monaco, Angela Carta, Francesco Taus, Lorena Torroni, Giuseppe Verlato, Stefano Porru

**Affiliations:** 1 Occupational Medicine Unit, University Hospital of Verona, 37134 Verona, Italy; 2 Section of Occupational Health, Department of Diagnostics and Public Health, University of Verona, 37134 Verona, Italy; 3 Section of Epidemiology and Medical Statistics, Department of Diagnostics and Public Health, University of Verona, 37134 Verona, Italy; 4 Unit of Forensic Medicine, Department of Diagnostics and Public Health, University of Verona, 37134 Verona, Italy

**Keywords:** Obstructive sleep apnea, Respiratory sleep disorders, STOP-BANG questionnaire, Healthcare workers, Occupational health

## Abstract

**Purpose:**

Obstructive sleep apnea (OSA) is a common respiratory sleep disorder, related to increased mortality, poor quality of life, and higher risk of work accidents and injuries. Studies on the risk of OSA (rOSA) among health workers (HW) are scant. The aims of this study were to investigate this issue in a large University Hospital and to assess the effectiveness of a screening program.

**Methods:**

The STOP-BANG questionnaire (SBQ) was sent via e-mail to the 5031 HW employed at the University Hospital of Verona. HW who completed the SBQ were classified at low, moderate, and high rOSA. HW at high rOSA were invited to undergo nocturnal polygraphy. The determinants of rOSA were studied by non-parametric Kruskal–Wallis test, Pearson's chi-squared, and multinomial logistic model.

**Results:**

Of 5031 HW, 1564 (31.1%) completed the online questionnaire. Responders with low, moderate, and high rOSA were 72.7%, 13.7%, and 13.6%. Male gender, older age, and higher body mass index (BMI) were significant predictors of high rOSA, as expected. Physicians had the lowest probability of being in the high-risk category. Polygraphy was performed in 64 subjects. The positive predictive value of the self-administered SBQ was 68.8% (95%C.I. 55.9–79.8%) but raised to 96.9% (95%C.I. 89.2–99.6%) when re-administered by medical staff.

**Conclusion:**

SBQ showed its effectiveness as a screening tool in detecting undiagnosed OSA in HW. Systematic screening for OSA in work settings could allow early diagnosis and treatment, reducing short- and long-term health effects of OSA.

## Introduction

Obstructive sleep apnea (OSA) is a common respiratory sleep disorder, characterized by partial or complete upper airway obstruction. As a result, it causes repetitive arousal, oxygen desaturation, and sympathetic activation. The severity of OSA is classified as mild, moderate, and severe, based on the number of the apnea/hypopnea index (AHI) (5–14, 15–29, > 30 events per hour, respectively) (Singh and Bonitati 2021). The estimated prevalence in the general population is very high and ranges largely, depending on the diagnosing criteria adopted by the different studies. A systematic review by Senaratna et al. (Senaratna et al. 2017) reported a value of 9–38% of subjects with at least 5 events per hour, and of 6 to 17%, considering moderate to severe OSA. Older age, male sex and obesity are the main determinants of higher prevalence (Senaratna et al. 2017). In agreement with these data, a systematic revision by Borsoi et al. (Borsoi et al. 2022) reported a prevalence of moderate–severe OSA of 9–27% in the Italian adult population, corresponding with 4–12 million subjects; however, only a small minority are diagnosed with OSA. Indeed, it is believed that among subjects affected by OSA, more than 90 percent of women and 80 percent of men are undiagnosed (Abrishami et al. 2010). Several reviews and meta-analyses investigated the long-term consequences of untreated OSA. Fu et al. (Fu et al. 2017) found a significant correlation between severe OSA and cardiovascular and all-cause mortality (Hazard Ratio—HR = 2.73; 95% CI = 1.94–3.85 and HR = 2.13; 95% CI = 1.68–2.68, respectively). Similar results were reported by Pan et al. (Pan et al. 2016) and Wang et al. (Wang et al. 2013). Furthermore, a dose–response association effect was shown. Indeed, a 10-unit increase in AHI resulted in a risk 17% higher for cardiovascular disease (Wang et al. 2013). On the other hand, OSA patients who are treated with continuous positive airway pressure significantly reduced these risks. In particular, compared to untreated subjects, patients who used Continuous Positive Airway Pressure (CPAP) had a lower risk of all-cause and cardiovascular mortality (HR = 0.66; 95% CI = 0.59–0.73). Comparing treated patients and controls, the HR for cardiovascular disease did not significantly differ, while HR for all-cause mortality persisted slightly greater in treated ones but still lower than in untreated (HR = 1.35 and HR 1.81, respectively) (Fu et al. 2017). OSA is also related to short-term effects. Indeed, it negatively impacts neurocognitive skills, leading to poor quality of life and worsening memory, attention, and vigilance (Guglielmi et al. 2015). As a consequence, undiagnosed OSA is related to work limitations. In particular, untreated subjects have a diminished ability to learn new tasks, and to concentrate, as well as higher rates of absenteeism and occupational accidents (Guglielmi et al. 2015; Garbarino et al. 2016; Chou et al. 2022). Healthcare workers (HW) are at high risk for sleep disorders, due to night shifts, long working hours, and work-related stress, resulting in a possible hazard to the safety of themselves and patients (Shaik et al. 2022). Indeed, a study by Hassani et al. (Hassani et al. 2015) investigated the association between occupational accidents and OSA among hospital staff, showing that HW at high rOSA had increased odds of having work-related injuries (OR = 2.74; 95% CI = 1.52–4.92). Although these data should be causes of concern for public health, studies investigating the prevalence of OSA among HW are few and the majority involved small groups (Geiger-Brown et al. 2013; Seyedmehdi et al. 2016; Aydın Güçlü et al. 2019; Alexandropoulou et al. 2019; Pascoe et al. 2020). Therefore, it should be a priority to assess the impact of this clinical condition in the healthcare sector to ensure the safety of both HWs and patients.

The gold standard for the diagnosis of OSA are polysomnography and the nocturnal polygraphy. However, these tests are very expensive and time-consuming (Abrishami et al. 2010). Low-cost and easy tools are needed to screen large populations, such as in working settings. Indeed, screening questionnaires showed good reliability and usefulness in these scenarios (Chen et al. 2021). In particular, several studies reported that SBQ had high sensitivity and methodological quality, both in sleep clinic patients and in general population (Abrishami et al. 2010; Luo et al. 2014; Chung et al. 2014, 2016).

This study aimed to assess the following:

- the prevalence of HW at low, moderate, and high rOSA in a large HW population belonging to an Italian University Hospital;
- the feasibility of a screening program targeted at the detection of undiagnosed OSA among HW by means of standardized methods.

## Materials and methods

### Study design, setting, and participants

The study was carried out from January 2020 to January 2021 by the Occupational Medicine Unit, with the collaboration of the Prevention and Protection Service of the University Hospital of Verona (AOUI VR).

The research was addressed to 5031 HW employed in AOUI VR at enrolling time. The study included an online screening (phase I), in which high-risk individuals were identified and invited to undergo a medical examination and a nocturnal polygraphy (phase II).

Ethical approval was received from the "Comitato Etico per la Sperimentazione Clinica delle province di Verona e Rovigo" (prot. N. 59,664 23/10/2019). Electronic informed consent was obtained from all subjects at phase I.

### Phase I

All the employees of the University Hospital of Verona received a questionnaire at their institutional email address, which included sociodemographic (sex, age, date of birth, and job title), clinical (height, weight, smoking habits, chronic diseases), and occupational (night shifts) items, and the STOP-BANG questionnaire (SBQ). Before filling in the questionnaire, participants were also asked to release informed consent. The number of night shifts was coded in three levels (0, 1–42, > 42), by splitting people with night shifts in two groups of equal size.

HW were classified into three categories, following the scoring of the STOP-BANG test designed by Chung et al. (2016):

- low rOSA
- moderate rOSA
- high rOSA

### Phase II

HW at high rOSA were invited to perform nocturnal polygraphy (Embletta® MPR Sleep System). Sleep tracks were analyzed through Embla RemLogic™ by trained medical staff. Mild, moderate, and severe OSA were diagnosed in patients who reached 5–14, 15–29, and ≥ 30 AHI, respectively (Singh and Bonitati 2021). Subjects who had mild OSA and a number of AHI < 5 in non-supine decubitus were treated with positional devices and repeated polygraphy. A pulmonological examination was recommended for HW with mild non-positional or moderate and severe OSA.

### Statistical analyses

Qualitative variables were reported using absolute and percentage frequencies, quantitative variables with asymmetric distribution through median and interquartile range (IQR).

Prevalence estimates were presented with a relative 95% confidence interval (CI) computed by the method of Clopper–Pearson.

The association between rOSA (low, moderate, and high) and potential determinants was studied by non-parametric Kruskal–Wallis test for quantitative variables and Pearson's chi-squared for categorical variables.

Determinants of OSA risk were further evaluated by a multinomial logistic model, where rOSA (low, moderate, high) was the response variable; job title (nurse, physician, technician, administrative, other); smoking habits (never, former, current smokers), and nasal polyposis/chronic sinusitis—the potential determinants; and gender, age (ten-year increase), and BMI—the potential confounders.

The agreement between responses to the online self-administered questionnaire and the questionnaire administered by the physician during the visit was evaluated by Cohen’s kappa coefficient. Analyses were performed using STATA statistical software, release 17 (StataCorp, College Station, TX, USA), and statistical significance was set at p < 0.05.

## Results

### Screening phase

Of 5031 HW, 1564 (31.1%) completed the online questionnaire. As shown in Table 1, responders comprised a larger proportion of women and nurses, and a lower proportion of physicians than non-responders. The association between response and shift work was not significant.

**Table 1:** Demographic and occupational characteristics of HW participating or not participating in the online screening survey

	Non-Responders n = 3467 (%)	On line Responders n = 1564 (%)	p-value
Sex			< 0.001
Women	2385 (68.8%)	1254 (80.2%)	
Men	1082 (31.2%)	310 (19.8%)	
Age, median (IQR)	51 (42, 57)	50 (43, 55)	0.054
Job Task			< 0.001
Administrative	408 (11.8%)	177 (11.3%)	
Nurse	1350 (38.9%)	736 (47.1%)	
Physician	657 (19.0%)	209 (13.4%)	
Technician	445 (12.8%)	185 (11.8%)	
Other health professional	607 (17.5%)	257 (16.4%)	
Night Shifts			0.439
0	2200 (63.5%)	977 (62.5%)	
1–24	669 (19.3%)	294 (18.8%)	
> 24	598 (17.2%)	293 (18.7%)	

P-values were computed by Pearson's chi-squared test for categorical variables and Wilcoxon rank-sum test for continuous variable (age)

Responders with low, moderate, and high rOSA were 1137 (72.7%), 215 (13.7%), and 212 (13.6%), respectively (Table 2). As expected, age and BMI increased with increasing rOSA, as well as the proportion of men and patients treated for arterial hypertension. Of note, these variables are all involved in risk definition.

**Table 2:** Relation between OSA risk and individual variables involved in risk definition

	Low risk n = 1137 (%)	Moderate risk n = 215 (%)	n = 212 (%) High risk
Sex			
F	993 (87.3%)	161 (74.9%)	100 (47.2%)
M	144 (12.7%)	54 (25.1%)	112 (52.8%)
Age, median (IQR)	48 (41, 54)	55 (52, 59)	53 (48, 58)
BMI, median (IQR)	22.5 (20.6, 24.8)	25.2 (22.8, 28.0)	27.7 (25.2, 31.6)
BMI			
Normal weight	858 (75.5%)	103 (47.9%)	50 (23.6%)
Overweight	215 (18.9%)	77 (35.8%)	93 (43.9%)
Obese	64 (5.6%)	35 (16.3%)	69 (32.5%)
Treated hypertension	51 (4.5%)	72 (33.5%)	84 (39.6%)

Current and past smokers were more prevalent in the moderate/high than low-risk group. As regards job tasks, the high-risk group comprised the largest proportion of administrative workers, while nurses represented more than half of the low-risk group. The proportion of shift work decreased from the low- to the high-risk group (Table 3).

**Table 3:** Main sociodemographic characteristics of the low-/moderate-/high-risk groups

	Low risk n = 1137 (%)	Moderate risk n = 215 (%)	High risk n = 212 (%)	*p*-value
Do you smoke cigarettes?				< 0.001
No	815 (71.7%)	133 (61.9%)	129 (60.8%)	
Yes	178 (15.7%)	38 (17.7%)	38 (17.9%)	
Former	144 (12.7%)	44 (20.5%)	45 (21.2%)	
Job Task				< 0.001
Administrative	111 (9.8%)	30 (14.0%)	36 (17.0%)	
Nurse	580 (51.0%)	79 (36.7%)	77 (36.3%)	
Physician	156 (13.7%)	25 (11.6%)	28 (13.2%)	
Technician	116 (10.2%)	34 (15.8%)	35 (16.5%)	
Other health professional	174 (15.3%)	47 (21.9%)	36 (17.0%)	
Night shifts				0.037
0	684 (60.2%)	146 (67.9%)	147 (69.3%)	
1–42	223 (19.6%)	35 (16.3%)	36 (17.0%)	
> 42	230 (20.2%)	34 (15.8%)	29 (13.7%)	

*P*-values were computed using Pearson's chi-squared test or Fisher's exact test

The prevalence of comorbidities was rather low in this relatively young population. Indeed, hypertension, nasal polyposis/chronic sinusitis, and asthma/Chronic obstructive pulmonary disease (COPD) affected > 5% of responders (13.2%, 8.9%, and 6.8%, respectively), while atrial fibrillation, diabetes, ischemic heart disease affected 1.3%, 1.0%, and 0.6%, respectively. The prevalence of all comorbidities considered was the highest in the high-risk group (Table 4).

**Table 4:** Comorbidities in the low-/moderate-/high-risk groups

	Low risk	Moderate risk	High risk	p-value
Type 2 diabetes mellitus	7 (0.6%)	1 (0.5%)	8 (3.8%)	0.001
Asthma/COPD	72 (6.3%)	14 (6.5%)	20 (9.4%)	0.25
Nasal polyposis and/or chronic sinusitis	69 (6.1%)	26 (12.1%)	44 (20.8%)	< 0.001
Ischemic heart disease	2 (0.2%)	2 (0.9%)	5 (2.4%)	0.001
Atrial fibrillation	9 (0.8%)	4 (1.9%)	7 (3.3%)	0.008

P-values were computed using Pearson's chi-squared test or Fisher's exact test

These findings were confirmed in multivariable analysis (Table 5). Male gender, older age, and higher BMI were significant predictors of high rOSA, as expected. Among the other factors, nasal polyps/chronic sinusitis (NP/CS) was the strongest predictor. With respect to subjects without NP/CS, HW reporting NP/CS presented twice risk of being in the OSA medium risk category and five-fold increased risk of belonging to the high-risk category. Regarding job task, the risk of being in the high-risk group for OSA was the highest in the administrative staff and the lowest among physicians. On the other hand, smoking habits had no significant association with rOSA (Table 5).

**Table 5:** Determinants of the OSA risk evaluated by a multinomial logistic model, using low-risk OSA as reference category. The effects were synthetized through the Relative Risk Ratios (RRR)

	Moderate risk of OSA			High risk of OSA	
	RRR (CI 95%)	*P* value		RRR (CI 95%)	*P* value
Job Task					
Administrative	1 (reference)			1 (reference)	
Nurse	0.9 (0.5–1.4)	0.600		0.6 (0.3–1.0)	0.076
Physician	0.4 (0.2–0.9)	0.019		0.2 (0.1–0.5)	< 0.001
Technician	1.4 (0.7–2.5)	0.292		0.8 (0.4–1.6)	0.522
Other health professional	1.3 (0.7–2.3)	0.361		0.9 (0.4–1.6)	0.650
Smoking Habits					
Never	Ref			Ref	
Former	1.4 (0.9–2.2)	0.108		1.5 (0.9–2.5)	0.095
Current smokers	1.3 (0.9–2.1)	0. 184		1.3 (0.8–2.2)	0.225
Nasal polyposis and/or chronic sinusitis					
No	Ref			Ref	
Yes	2.2 (1.3–3.8)	0.003		5.2 (3.1–8.8)	< 0.001
Sex					
Female	Ref			Ref	
Male	3.33 (2.1–5.2)	< 0.001		14.8 (9.4–23.2)	< 0.001
Age per 10-year increase	3.8 (3.0–5.0)	< 0.001		2.3 (1.7–2.9)	< 0.001
BMI	1.1 (1.1–1.2)	< 0.001		1.3 (1.2–1.4)	< 0.001

### Clinical phase

Sixty-four HW, out of 212 invited, participated in the clinical phase. Of these, 20 (31%) were negative at polygraphy examination, 20 (31%) presented positional OSA and 24 (38%) had non-positional OSA. Consequently, the positive predictive value (PPV) of the online questionnaire in detecting OSA (mild, moderate and severe) was 68.8% (95% confidence interval = 55.9–79.8%). The PPV of the SBQ largely improved when the questionnaire was filled in by the physician in the occupational clinic (96.9%, 95% CI 89.2–99.6%).

## Discussion

Our study investigated the prevalence of HW at low, moderate, and high rOSA and the feasibility of a screening program targeted at the detection of undiagnosed OSA in a population of over 5,000 HW belonging to a large Italian University Hospital.

The proportion of response to the online questionnaire was similar in the present study and in a previous one, simultaneously carried out in the same University Hospital (31 vs 34%), and higher than others targeting HW populations (Spiteri et al. 2023). In agreement with the previous study, also the percentage of response among women and nurses was significantly higher than among men and other professional groups. No significant association was detected between the percentage of response and night shifts.

Our study showed that 13.6% of Verona HW were at high rOSA. Interestingly, this value falls in the lower range of diagnosed OSA prevalence (9–38%), reported for the general population aged > 18 years. It should be reminded, however, that the prevalence estimated for the working age classes (20–65 years old) is lower than the prevalence reported for elderly people (Senaratna et al. 2017).

The prevalence of rOSA, found in studies involving only HW, varied widely as a consequence of different characteristics of the enrolled populations and different methods for identifying at-risk individuals. Pascoe et al. (Pascoe et al. 2020) found a very high proportion of individuals at high rOSA (37%) in a population of 2851 American caregivers using the STOP questionnaire. Accordingly, 7% of this population even had a prior diagnosis of OSA. However, as acknowledged by the authors themselves, the percentage of respondents was very low (about 6%) in that study, so that nonresponse bias cannot be excluded (Verlato et al. 2010). In particular, the lack of sociodemographic data did not allow determining the age characteristics of the sample. Hence, considering the high number of subjects with a prior diagnosis of OSA (7%), it could be assumed that the HW who participated in this study were older than non-responders.

Three studies, dealing with OSA risk among HW, adopted the Berlin Questionnaire rather than SBQ (Seyedmehdi et al. 2016; Aydın Güçlü et al. 2019; Alexandropoulou et al. 2019). Seyedmehdi et al. (Seyedmehdi et al. 2016) reported a prevalence of 6.9% among a population of 715 HW. The lower prevalence as compared to our study could be explained by the younger age (mean = 33.5 years) beside our study (48.7 years). However, another study performed on 604 HW of similar age (median = 34.9 years) found a prevalence of rOSA (15.1%) comparable to our results (13.6%) (Aydın Güçlü et al. 2019). Hence, age alone cannot fully explain variability across studies, which is likely influenced by several other factors. For instance, Alexandropoulou et al. (Alexandropoulou et al. 2019) found a very high prevalence of rOSA in a Greek nursing staff population (20.5% of 444 participants), mostly overweight or obese. Indeed, the median BMI among participants was 27.2 kg/m^2^, significantly higher than the value of 23.4 kg/m^2^, found in our study population, and the higher BMI could possibly explain the higher prevalence in rOSA.

In our study, night shifts were associated with a lower rOSA. Paciorek et al. (Paciorek et al. 2011) investigated the effect of the night shifts in a group of 10 workers affected by OSAS, and found that the severity of the disease worsened after working at night. Other studies confirmed that sleep disorders can be triggered by night shifts (Yazdi et al. 2014; Pascoe et al. 2020). On the other hand, a systematic review by Yang et al. (Yang et al. 2021), exploring the association between shift work and OSA, reported a small, non-significant increase in the rOSA in night shift workers (RR = 1.05; 95% CI 0.85–1.30), so they concluded that the results were inconclusive. In our opinion, the negative association between night shifts and rOSA could be attributed to reverse causation, i.e., an healthy worker effect, as subjects with symptoms related to OSA are often excluded from night shifts.

During the clinical phase, we performed 64 polygraphies. Of these, 40 were positive, showing that SQB achieved a rather good PPV (68.8%) for mild to severe OSA. An even higher value was estimated by meta-analysis performed by Abrishami et al. (2010), which yielded a PPV of 84% for AHI ≥ 5 in patients without a history of sleep disorders. Restricting the outcome definition to moderate/severe or only severe OSA the value raised to 93% and 100%, respectively. PPV is largely influenced by the research setting, as it increases with the prevalence of the disease. However, also sensitivity, which is independent of disease prevalence from a theoretical point of view (Altman 1991) but not in clinical practice (Mark 2005), was quite good. Sensitivity was 73% and 97.6% in meta-analyses of studies on the general population or sleep clinic patients, respectively (Amra et al. 2018; Chen et al. 2021). High sensitivity (83.6%) was reported also in a study involving only sleep clinic patients (Luo et al. 2014; Amra et al. 2018). These data show that the PPV and the sensitivity of SBQ improve when tests are administered to high-prevalence populations, but they remain reliable even for healthy working populations.

Our study showed that the PPV increased from 68% to 96.9% when the questionnaire was administered by trained medical staff. This finding seems to suggest that screening for high rOSA in the workplace should be performed by occupational physicians during the periodic health surveillance, leading to an improvement of the test effectiveness, as well as health promotion in work settings.

This study has some limitations. First of all, the low response rate to the on-line questionnaire (31%). However, as discussed before, it is similar or even higher than in previous studies among HW, suggesting that greater adhesion rates are difficult to achieve in this population. Moreover, only a low number of HW underwent polygraphy (64 out of 212). Indeed, it should be underlined that adherence to the clinical phase was influenced by the COVID pandemic during 2020. Finally, sociodemographic and clinical data are self-reported, affecting their reliability.

Our study has also several strengths. To the best of our knowledge, our research is the second largest by the number of HW involved and it is the largest that has clinical and sociodemographic data available for all the HW included. Furthermore, using of a standardized questionnaire let us to achieve comparable and repeatable results. Moreover, although most of the studies that investigated the rOSA among HW used the Berlin Questionnaire, we chose the SBQ because it was validated in work settings, such as commercial drivers, had the highest sensitivity, and was the easiest and fastest to fill in (Abrishami et al. 2010; Amra et al. 2018; Lonia et al. 2020; Chen et al. 2021). A further strength regards the instrumental confirmation performed in HW classified at high rOSA by SBQ; this approach allowed us to calculate PPV of both self and trained medical staff administered SBQ.

## Conclusion

Our results suggest that systematic screening for OSA among HW could detect subjects at high rOSA to be referred to pneumological evaluation, to avoid short- and long-term risks for the safety and the health of both HW and patients. To improve the positive PPV and reduce the number of unnecessary polygraphies, it is recommended that subjects, who resulted at high risk according to the self-administered SBQ, repeat the test under the supervision of trained medical staff before undergoing the instrumental examination.

## Data Availability

The data that support the findings of this study are not openly available due to reasons of sensitivity and are available from the corresponding author upon reasonable request.
